# A Trying Time: Problematic Internet Use (PIU) and its association with depression and anxiety during the COVID-19 Pandemic

**DOI:** 10.1186/s13034-022-00479-6

**Published:** 2022-06-23

**Authors:** Sinduja Lakkunarajah, Keisha Adams, Amy Y. Pan, Melodee Liegl, Mandakini Sadhir

**Affiliations:** 1 Department of Pediatrics, Division of Adolescent Medicine, Medical College of Wisconsin Affiliated Hospitals, 999 N 92nd Street, Suite 460, Milwaukee, WI 53226 USA; 2 Present Address: Division of Adolescent Medicine, University of South Florida Health Morsani College of Medicine, 2 Tampa General Circle, Suite 5056, Tampa, FL 33606 USA; 3 Department of Pediatrics, Medical College of Wisconsin, Quantitative Health Sciences, 8701 Watertown Plank Road, Milwaukee, WI 53226 USA; 4 Department of Pediatrics, Kentucky Children’s Hospital, Suite L407, Lexington, KY 40536 USA

**Keywords:** Problematic Internet Use, PRIUSS, GAD-7, Anxiety, Depression, PHQ-9A, COVID-19

## Abstract

**Background:**

The prevalence of problematic Internet use (PIU) among adolescents and young adults (AYA) was approximately 9–11% before the COVID-19 pandemic. The purpose of this study was to determine the prevalence of PIU among AYAs (especially younger adolescents) during the COVID-19 pandemic using the Problematic and Risky Internet Use Screening Scale (PRIUSS). Additionally, we examined the relationship between PIU, depression and anxiety among AYAs during the same period.

**Methods:**

A descriptive-analysis survey study was completed over a 6-month period from January 4, 2021, to June 30, 2021. It was conducted at a tertiary care Adolescent Medicine Clinic with AYAs age 12–21. The PRIUSS screened for PIU, the PHQ-9A [Patient Health Questionnaire-9A] screened for depression, and the GAD-7 [General Anxiety Disorder-7] screened for generalized anxiety. Fisher’s exact test, the Mann–Whitney test and Spearman correlations were performed.

**Results:**

A positive PRIUSS score was observed in 18% of the 447 participants. Of these participants, 44% had a pre-existing diagnosis of depression, 39% had a pre-existing diagnosis of anxiety and 29% had a pre-existing diagnosis of depression and anxiety. There was a positive correlation between PRIUSS, PHQ-9A and GAD-7 total scores. A higher PRIUSS score was associated with a higher PHQ-9A and GAD-7 score (p < 0.001). There was also a positive correlation between a positive PRIUSS score and a pre-existing diagnosis of depression (p < 0.001).

**Conclusions:**

This study showed a higher prevalence of PIU during the COVID-19 pandemic using the PRIUSS. In addition, a positive correlation between PRIUSS scores and pre-existing diagnosis of depression, positive GAD-7 and PHQ-9A scores was noted. In conclusion, medical providers should consider screening for PIU in AYAs with positive mental health screens.

There are an estimated 2 billion internet users worldwide, and the majority are adolescents and young adults (AYAs) [1]. In the US, approximately 93% of AYAs use the internet for educational, professional and social purposes [2–4]. Its use has increased over time such that problematic internet use (PIU) behaviors have been noticed [2]. The current consensus on the definition of PIU is internet use that is risky, excessive or impulsive, is associated with adverse life consequences (physical, emotional, social or functional impairment) and is not associated with a primary psychiatric diagnosis [5–7].

Prior to the COVID-19 pandemic, the global prevalence of PIU among adolescents was approximately 2–11%, and among American adolescents, it was approximately 7–11%. These studies focused on college-aged AYAs and adults [1, 8, 9]. There are limited studies that look at PIU in younger Americans. One study conducted by Liu et al. [6], observed a lower prevalence of PIU of approximately 4% in Connecticut high school aged students [6]. The significant differences in prevalence ranges noted in these studies are likely due to the different screening tools used to assess PIU in the studies, such as the Young Diagnostic Questionnaire (YDQ) for Internet Addiction, the Internet Addiction Tool (IAT) and the Problematic and Risky Internet Use Screening Scale (PRIUSS) [10]. The PRIUSS was developed and refined in the US and studied in college-aged AYAs. The prevalence of PIU, prior to the COVID-19 pandemic, using the PRIUSS was approximately 9–11% and is currently the only validated screening tool for the pediatric population [10–12]. The use of the PRIUSS to determine the prevalence of PIU among younger American adolescents during the COVID-19 pandemic is unknown.

Studies prior to the COVID-19 pandemic have shown that PIU had a negative impact on mental health, such as depression and anxiety [3, 7, 13]. The estimated baseline lifetime prevalence of anxiety disorders and major depression in American adolescents is approximately 32% and 13%, respectively [14]. The COVID-19 pandemic has been associated with stress and times of isolation, and it is presumed that individuals may have engaged in behaviors that helped alleviate stress, whether it is substance use, video games, increased social media presence, or other activities online [15]. These behaviors could have potentially worsened depression, anxiety and psychological well-being [15]. Given the high baseline prevalence of these mental health conditions, there is a likelihood that these conditions worsened during the COVID-19 pandemic. A recent retrospective cohort study performed in the US with 12–23-year-old participants showed that those with higher PIU screening scores (using the PIU-SF-6 screening tool) were more likely to have higher PHQ-8 (Patient Health Questionnaire-8) and GAD-7 (General Anxiety Disorder-7) scores [16]. However, there is limited research done on the association of PIU and mental health using the PRIUSS, PHQ-9A (Patient Health Questionnaire-9A) and GAD-7.

The study’s first aim was to determine the prevalence of PIU among American AYAs during the COVID-19 pandemic using the PRIUSS. The second aim was to determine the relationship between PIU and depression and anxiety among American AYAs during the COVID-19 pandemic using the PHQ-9A and GAD-7. The focus was on younger adolescents, as there is limited research on PIU in this population. The hypothesis was that a higher prevalence of PIU would be noted during the COVID-19 pandemic using the PRIUSS and there would be a positive correlation between the total PRIUSS scores and PHQ-9A and GAD-7 scores.

## Methods

### Study design and participants

A descriptive-analysis survey study was designed to address the study objectives. The study was conducted over a 6-month period from January 4, 2021, to June 30, 2021, at an Adolescent Medicine Specialty Clinic, which was located at a Midwestern tertiary care Children’s Hospital. The Adolescent Medicine clinic provided care regarding reproductive health, eating disorders and care for patients with general mental health conditions. Eligible participants were adolescents aged 12–21 years old who were able to read and fill out the screening tools and questionnaire independently (without the assistance of a parent/guardian or medical provider) and, had access to an electronic device with internet services. The screening tools used included the PHQ-9A for depression, GAD-7 for anxiety, and PRIUSS for PIU. A power calculation was performed for the study based on the correlation between the PHQ-9A total score and the PIU total score. With a sample size of 1000, we will be able to detect a correlation of 0.1 with an alpha of 0.05 and a power of 90%.

### Screening tools and participant questionnaire

The PHQ-9A is a 9-item depression screening tool that was modified from the PHQ-9 for adolescents, where it also assessed suicide risk. Each item is scored from 0 (not at all) to 3 (nearly every day) with a score range of 0–27 and has a sensitivity of 75% and specificity of 94% [17, 18]. A positive PHQ-9A score is 10 and higher; however, a score of 5–9 is classified as mild depression. For the purpose of this study, a score of 5 or higher was considered positive. The GAD-7 is a 7-item screening tool that assesses worry and anxiety [19, 20]. Each item is scored from 0 (not at all) to 3 (nearly every day) with a score range of 0–21. A positive GAD-7 score is 10 and higher, with a sensitivity of 89% and a specificity of 82% [20]. However, a score of 5–9 is classified as mild anxiety, and for the purpose of this study, a score of 5 or higher was considered positive. The PRIUSS is an 18-item screening tool for PIU with 3 subscales for social impairment, emotional impairment and risky/ impulsive internet use. Each item is scored on a Likert scale from 0 (never) to 4 (very often), and a positive score is 25 and higher with a sensitivity of 80% and specificity of 79% [21]. In addition to the screening tools administered, participants were given a short questionnaire. The questionnaire asked about the participant’s demographics (age, gender identity, race/ethnicity), their medical record number (to ensure that there was only one unique response for each participant during the data collection period), reason for the visit to clinic (reproductive health concerns, eating disorder concerns or mental health concerns) and pre-existing diagnosis of depression and/or anxiety. A pre-existing diagnosis of depression and/or anxiety was defined as a participant already having an established diagnosis of the condition by a medical provider (either made in this clinic or at another clinic) and was seen by a medical provider for treatment of the condition. Participants then indicated if they saw a therapist and/or a psychiatrist for their mental health and if they received medication.

Participants completed the survey while they waited to see the medical provider. The surveys were collected by the participant’s medical provider and given to the study investigator after any concerns were addressed. As part of the current standard of care in the clinic, any positive scores on the screening tools were addressed by the medical provider with the participant and parents and were provided with appropriate referrals, resources and follow-up. A social worker was also available in the clinic if the participant required a safety plan or an escalation in care was required regarding their mental health and/or their safety. All responses were recorded in REDCap. The study was approved by the Institutional Review Board of Children’s Wisconsin.

## Analysis

Data were reported as N (%) or median and interquartile range (IQR). Fisher’s exact test was used to compare categorical variables, while the Mann–Whitney-Wilcoxon test was used to compare continuous variables. Scatterplots were generated, and Spearman correlation coefficients were calculated to examine the relationship between PRIUSS total score and PHQ-9A total score or GAD-7 total score. These relationships were further investigated by a general linear model adjusting for demographics and potential confounders (age, sex, race/ethnicity, depression status, anxiety status). Multiple logistic regression was also performed to examine the association between the risk of PIU (PRIUSS score < 25 vs PRIUSS score ≥ 25) and PHQ-9A total score or GAD-7 total score. Data were log transformed to improve fit. The trend in proportions of participants with risk for PIU over different severities of depression (determined by PHQ-9A score ranges) or anxiety (determined by GAD-7 score ranges) were assessed by the Cochran-Armitage test. A p-value of < 0.05 was considered significant. SPSS version 28.0 (Chicago, Illinois, USA) and SAS version 9.4 (SAS Institute Inc., Cary, NC) were used for statistical analyses.

## Results

### Participants

There were a total of 447 unique participant responses (Table 1). Ninety-six percent identified themselves as female, and the median age was 16 years (yr) (IQR, 15–17). Twenty-two percent were middle school aged (12–14 yr), 71% were high-school aged (15–18 yr) and 7% were young adults (19–21 yr). Ninety-five percent were enrolled in school, with the majority learning virtually. All had access to one or more electronic devices (95% smartphone access, 39% tablet access and 91% computer/ laptop access).

**Table 1:** Demographics for the overall participant population and further divided into risk of PIU

	Overall		No risk for PIU (score < 25)		Risk for PIU (score≥ 25)		P-value
	N Eval	N (%) or Median (IQR)	N Eval	N (%) or Median (IQR)	N Eval	N (%) or Median (IQR)	
**Age, years**	447		368		79		0.60
12–14		100 (22)		82 (22)		18 (23)	
15–18		317 (71)		259 (71)		58 (73)	
19–21		30 (7)		27 (7)		3 (4)	
**Gender**	447		368		79		0.032
Male		12 (3)		9 (2)		3 (4)	
Female		427 (96)		355 (97)		72 (91)	
Other		8 (1)		4 (1)		4 (5)	
**Race**	446		367		79		0.17
Caucasian		234 (53)		94 (53)		40 (51)	
African American		112 (25)		92 (25)		20 (25)	
Hispanic		64 (14)		56 (15)		8 (10)	
Mixed		36 (8)		25 (7)		11 (14)	
**Enrolled in school**	447	424 (95)	368	349 (95)	79	75 (95)	0.99
**Type of schooling**	424		349		75		0.45
In-person		161 (38)		136 (39)		25 (33)	
Virtual		208 (49)		166 (48)		42 (56)	
Hybrid		55 (13)		47 (13)		8 (11)	
**Employed**	447	169 (38)	368	139 (38)	79	30 (38)	0.99
**Electronic devices used**^a^							
Smart phone	447	425 (95)	368	350 (95)	79	75 (95)	0.99
Tablet	447	172 (39)	368	140 (38)	79	32 (41)	0.70
Computer/laptop	447	406 (91)	368	331 (90)	79	75 (95)	0.20

^a^The participant may have used more than one device

### Mental health

Of the total sample, 33% had a pre-existing diagnosis of anxiety, 29% had a pre-existing diagnosis of depression and 22% had a pre-existing diagnosis of anxiety and depression. Sixty percent of the participants did not have an existing diagnosis of anxiety and depression. Upon review of the screening tools, 59% had a positive GAD-7 screen (score ≥ 5), of which 56% did not have a pre-existing diagnosis of anxiety; 58% had a positive PHQ-9A screen (score ≥ 5), of which 58% did not have a pre-existing diagnosis of depression (Table 2).

**Table 2:** Pre-existing mental health condition and the respective screening tool scores for all the study participants

	N Eval	N (%) or Median (IQR)
**Anxiety**		
Pre-existing diagnosis of anxiety	447	
No		299 (67)
Yes		148 (33)
Patients with diagnosis of anxiety		
See a therapist	148	88 (60)
See a psychiatrist	147	53 (36)
Taking medication for anxiety	148	100 (68)
GAD-7 positive screen for anxiety	262	
No pre-existing diagnosis of anxiety		146 (56)
Pre-existing diagnosis of anxiety		116 (44)
**Depression**		
Pre-existing diagnosis of depression	447	
No		319 (71)
Yes		128 (29)
Patients with diagnosis of depression		
See a therapist	128	82 (64)
See a psychiatrist	128	60 (47)
Taking medication for depression	128	88 (69)
PHQ-9A, positive screen for depression	259	
No pre-existing diagnosis of depression		150 (58)
Pre-existing diagnosis of depression		109 (42)
Pre-existing diagnosis of anxiety and depression	447	97 (22)
**GAD-7**	447	
Minimal anxiety (score 0–4)		185 (41)
Mild anxiety (score 5–9)		92 (21)
Moderate anxiety (score 10–14)		91 (20)
Severe anxiety (score 15–21)		79 (18)
**PHQ-9**	447	
No or minimal depression (score 0–4)		188 (42)
Mild depression (score 5–9)		115 (26)
Moderate depression (score 10–14)		68 (15)
Moderately severe depression (score 15–19)		51 (11)
Severe depression (score 20–27)		25 (6)

### Problematic internet use

A positive PRIUSS score (score ≥ 25) was seen in 18% of the participants. Of these, 44% had a pre-existing diagnosis of depression, 39% had a pre-existing diagnosis of anxiety and 29% had a pre-existing diagnosis of depression and anxiety (Table 3).

**Table 3:** Looks at the relationship between PRIUSS, GAD-7 and PHQ-9A scores and pre-existing diagnosis of Anxiety and Depression

	No Risk for PIU (score< 25)		Risk for PIU (score ≥ 25)		P-value
	N Eval	N (%) or Median (IQR)	N Eval	N (%) or Median (IQR)	
Anxiety	368		79		0.24
No		251 (68)		48 (61)	
Yes		117 (32)		31 (39)	
Depression	268		79		< 0.001
No		275 (75)		44 (56)	
Yes		93 (25)		35 (44)	
Anxiety AND Depression	368		79		0.10
No		294 (80)		56 (71)	
Yes		74 (20)		23 (29)	
PHQ-9A	368	5 (2–10)	79	12 (8–17)	< 0.001
No or minimal depression (score 0–4)		179 (49)		9 (12)	< 0.001
Mild depression (score 5–9)		95 (26)		20 (25)	
Moderate depression (score 10–14)		46 (13)		22 (28)	
Moderately severe depression (score 15–19)		35 (9)		16 (20)	
Severe depression (score 20–27)		13 (3)		12 (15)	
GAD-7	368	5 (2–11)	79	14 (9–17)	< 0.001
Minimal anxiety (0–4)		175 (48)		10 (12)	< 0.001
Mild anxiety (5–9)		82 (22)		10 (12)	
Moderate anxiety (10–14)		69 (19)		22 (29)	
Severe anxiety (15–21)		42 (11)		37 (47)	

Of the 268 participants who did not have a pre-existing diagnosis of depression and anxiety, 13% (n = 36) had a positive PRIUSS score, of which 81% (n = 29) had a positive GAD-7 screen (7 participants with mild anxiety, 9 participants with moderate anxiety and 13 participants with severe anxiety), 83% (n = 30) had a positive PHQ-9A screen (12 participants with mild depression, 10 participants with moderate depression, 4 participants with moderately severe depression and 4 participants with severe depression) and 75% (n = 27) had positive GAD-7 and PHQ-9A screens.

**Fig. 1:**
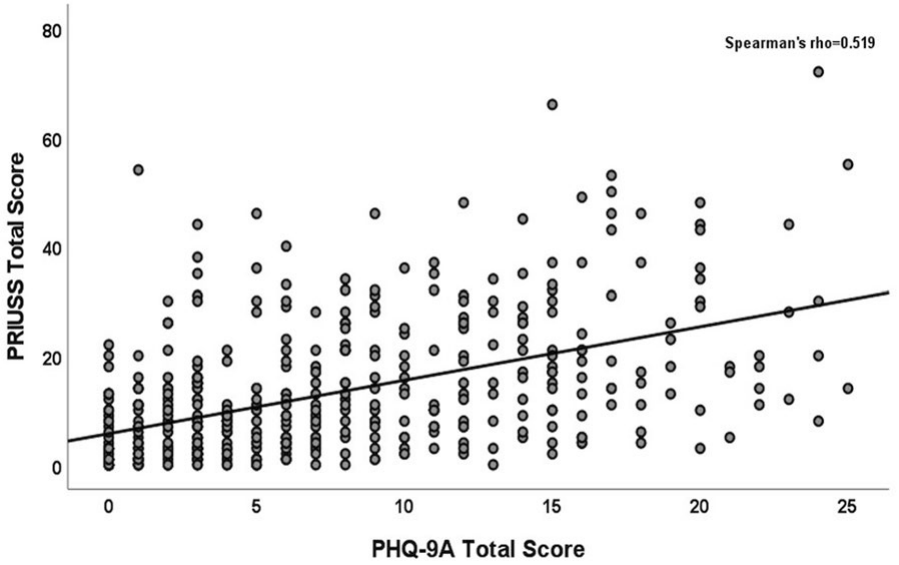
PRIUSS scores in relation to PHQ-9A scores for all study participants

**Fig. 2:**
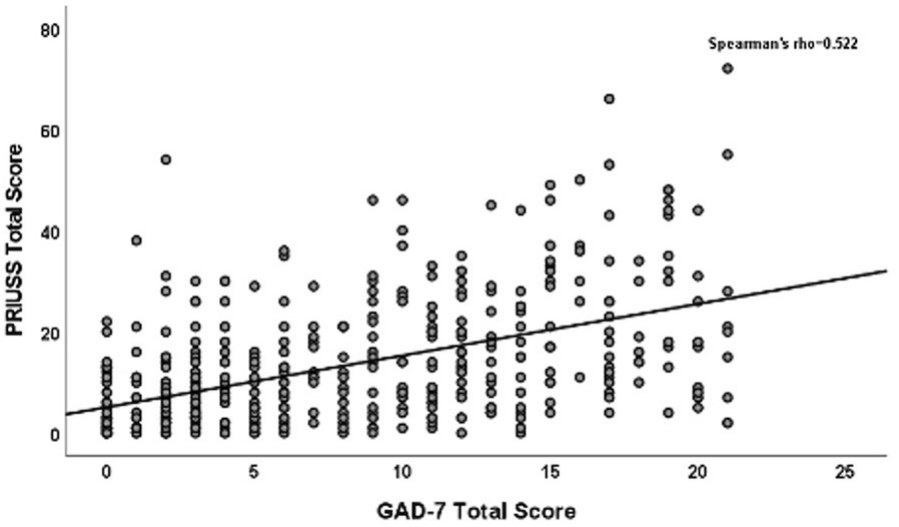
PRIUSS scores in relation to GAD-7 scores for all study participants

There were 97% of those who identified as female in the no risk for PIU group versus 91% in the risk for PIU group (p = 0.032). There were also more participants without a pre-existing diagnosis of depression in the no risk for PIU group (75%) versus those in the risk for PIU group (56%), p < 0.001. There were no significant differences in the risk and no risk groups for PIU when comparing age, ethnicity/race, attending schooling and whether they had a pre-existing diagnosis of anxiety (Tables 1–3). There was a positive correlation between PHQ-9A and GAD-7 scores and the PRIUSS total score (p < 0.001) (Figs. 1 and 2). Furthermore, after adjusting for demographics and potential confounders, the PHQ-9A total score or GAD-7 total score was found to be significantly associated with the PRIUSS total score. We expect to see an 8.7% increase in PRIUSS total score with a one-unit increase in PHQ-9A total score (p < 0.0001) and an 8.5% increase with a one-unit increase in GAD-7 total score (p < 0.0001). The PHQ-9A total score and GAD-7 total score were associated with an increased risk for PIU, with adjusted odds ratios and 95% confidence intervals of 1.14 (1.09, 1.20), p < 0.0001 and 1.18 (1.12, 1.24), p < 0.0001, respectively.

## Discussion

To our knowledge, this study is the first to examine the prevalence of PIU in younger adolescents during the COVID-19 pandemic using the PRIUSS, and to assess the association of PIU with depression and anxiety using the PRIUSS, PHQ-9A and GAD-7 scores respectively.

The prevalence of PIU using the PRIUSS in our study was 18%, which was higher than the 9–11% seen prepandemic among American college-aged students in other studies [12]. The prevalence in our study sample could have been higher due to COVID-19 pandemic-related factors, such as decreased in-person social interactions (mask mandates, school events cancelled, stay-at-home order), stress related to the COVID-19 pandemic, or increased free time associated with the pandemic, which could have resulted in engaging in unhealthy internet usage. Interestingly, in this study, no association was found between virtual format of schooling and PIU in contrast to other studies that showed some association [3, 22]. Mohammadkhan et al. and Twenge noted a higher risk for internet addiction given the increasing amount of internet use for educational and entertainment needs (texting, gaming, social media) [3, 22].

Our study is the first to our knowledge to evaluate PRIUSS scores and their association with PHQ-9A and GAD-7 scores in this population during the COVID-19 pandemic. A positive PRIUSS score positively correlated with PHQ-9A scores and GAD-7 scores. This can be due to worsening mental health during the COVID-19 pandemic and the COVID-19 pandemic-related factors mentioned above. This study also showed that those with a pre-existing diagnosis of depression had a positive correlation with a positive PRIUSS, which has been seen in other studies that used different screening tools for PIU, such as the study done by Restrepo et al. [7]. Our study did not show an association between a positive PRIUSS and those with a pre-existing diagnosis of anxiety. However, other studies have shown that there is a positive correlation between a positive screen for PIU and social anxiety [7, 23, 24]. Our study used a screening tool that screened for generalized anxiety disorder only, whereas other screening tools can evaluate for other anxiety disorders, such as social anxiety. Social anxiety was likely associated with PIU in other studies, as individuals may have engaged in risky online behaviors such as dating or befriending strangers. With the global rise in mental health concerns in the AYA population, there can be engagement in risky internet behaviors that can lead to PIU. Those with depression or social anxiety may engage in these behaviors to help compensate for the poor or lack of real-life relationships, as it can be easier to navigate and there is anonymity [7, 25].

Finally, our study showed that some participants who did not have a pre-existing diagnosis of depression and/or anxiety had a positive PHQ-9A and/or GAD-7 screen, indicating an undiagnosed mental health condition. Prior to the COVID-19 pandemic, The National Institute of Mental Health reported that the prevalence of at least one major depressive episode among American adolescents was 15.7%, and 31.9% of American adolescents had an anxiety disorder of some type [26, 27]. A meta-analysis by Racine et al. showed that during the COVID-19 pandemic, the prevalence of depressive symptoms and anxiety symptoms were 25.2% and 20.5%, respectively, which almost doubled from prior to the pandemic [28]. Many studies have shown that PIU negatively impacts social relationships and professional endeavors, as it leads to lower reported life satisfaction and well-being, lower reported happiness, feeling lonely and feeling socially isolated [2, 7, 22]. Hence, it is important that medical providers consider screening patients for PIU, especially in those with an underlying mental health diagnosis or those who have a positive mental health screen. The different ‘waves of the COVID-19 pandemic’ that have resulted in return to isolation or prolonged isolation, emphasizes the importance for ongoing screening.

There were several limitations to this study. First, the sample size was not met, which was due to eligible participants not completing all elements of the surveys or not having the predicted volume of eligible participants. Second, the study participants were largely female and Caucasian, which limited the generalizability of our findings. Additional studies need to focus on a more sociodemographic diverse population and include males. In addition to the demographic make-up of the participants, another limitation was that the participants were recruited from the clinic. Participants presenting to a clinic location can be concerned about the underlying reason or complaint that resulted in them having to make the visit to the clinic. The complaint related to the clinic may be negatively impacting the participant’s mental health as oppose to PIU. Therefore, recruiting participants from a non-clinical setting would eliminate this confounding variable. Lastly, this study only assessed PIU’s association with generalized anxiety using the GAD-7. Other screening tools, such as the SCARED, screen for different types of anxiety disorders (ie. school avoidance, panic disorder, separation anxiety, etc.). Therefore, our study only correlated PIU to GAD and not other anxiety disorders.

The next steps will be to continue to monitor the status of the COVID-19 pandemic and its association with PIU using the PRIUSS and mental health and what changes might be seen when the pandemic is over. Additionally, data regarding the association between PIU and anxiety is limited. It would be worthwhile to explore this using an anxiety screening tool such as the SCARED that screens for multiple anxiety disorders versus generalized anxiety only. Finally, as previous studies assessed socioeconomic status and family structure’s impact on internet addiction, we can investigate the impact of these factors on PIU using the PRIUSS and mental health [3, 29].

## Conclusion

In conclusion, this study showed a higher prevalence of PIU in AYAs (including a larger younger adolescent population) during the COVID-19 pandemic using the PRIUSS. In addition, an association between PIU, depression and anxiety using the PRIUSS, PHQ-9A and GAD-7 respectively, was noted. Finally, this study demonstrated the importance of medical providers screening for mental health conditions in general, as several participants were undiagnosed. More studies are needed to better understand the impact of PIU on the mental health of adolescents and young adults. This will facilitate the creation of meaningful resources and support for patients and families.

## Data Availability

All data generated or analyzed during this study are included in this published article.
